# Hematological Features in Sheep with IgG and IgM Antibodies against *Borrelia burgdorferi sensu lato*

**DOI:** 10.3390/pathogens10020164

**Published:** 2021-02-04

**Authors:** Labrini V. Athanasiou, Victoria M. Spanou, Eleni G. Katsogiannou, Panagiotis D. Katsoulos

**Affiliations:** 1 Department of Medicine, Faculty of Veterinary Medicine, University of Thessaly, 43100 Karditsa, Greece; vispanou@uth.gr (V.M.S.); elkatsog@uth.gr (E.G.K.); 2 Clinic of Farm Animals, Faculty of Veterinary Medicine, Aristotle University of Thessaloniki, 54627 Thessaloniki, Greece; katsoulo@vet.auth.gr

**Keywords:** anemia, *Borrelia*, Greece, hematology, serology, sheep, thrombocytopenia, tick-borne, zoonotic disease

## Abstract

Exposure of sheep to *Borrelia* *burgdorferi sensu* *lato* (*s.I.*) complex, the causative agent of Lyme borreliosis (LB), has been reported in tick-abundant areas worldwide, while no data have been reported in Greece. The aim of the study was to identify the hematological alterations in sheep with seropositivity against *Borrelia burgdorferi* (*s.I.*). Blood samples were obtained from 318 tick infested sheep for blood analysis and serological determination of IgG and IgM antibodies against *B. burgdorferi* by indirect immunofluorescence antibody (IFA) assay after exclusion of endo-ectoparasites and other tick-borne infections. A total number of 162 sheep met the inclusion criteria, allocated in four groups based on the presence or absence of IgG and/or IgM; sheep found negative for IgM and IgG (Group A), positive for IgM (Group B), positive for both IgM and IgG (Group C) and positive for IgG (Group D). Anemia, thrombocytopenia and normal or decreased leukocyte count, mainly due to lymphopenia were the main hematological features observed in seropositive sheep. The presence of these features raises the suspicion of *Borrelia* infection in tick infested sheep. The seropositivity of 23.58% in sheep raises concerns of *Borrelia* circulation, especially in rural areas and potential risk of transmission to humans.

## 1. Introduction

Lyme disease or Lyme borreliosis (LB) is a tick-borne disease with zoonotic potential. The etiologic agent is *Borrelia burgdorferi sensu lato* (*s.I.*) complex, helical-shaped bacteria belonging to the spirochaete phylum [1]. The complex consists of at least 21 genospecies to date [2], while mainly three of them, *Borrelia burgdorferi sensu stricto*, *Borrelia afzelii* and *Borrelia garinii* are of public health importance [3,4]. Transmission to humans and animals occurs by the bites of infected tick vectors of the genus *Ixodes*. In Europe the tick species of concern is *Ixodes ricinus*, while ticks of *Ixodes scapularis* and *Ixodes pacificus* species [5] are the main tick species in the eastern and in the northwestern United States, respectively [6].

Ticks infesting animals have been reported in different studies in Greece. In a study, ticks collected from goats, sheep, cattle and dogs belonged to five genera: *Ixodes* (*I. ricinus*, *I. gibbosus*), *Rhipicephalus* (*R. bursa, R. turanicus*, *R. sanguineus*), *Hyalomma* (*H. m. marginatum*), *Boophilus* (*B. annulatus*) and *Dermacentor* (*D. marginatus*) [7]. Moreover, seven different tick species were identified on sheep in a descending row including *D. marginatus*, *Haemaphysalis parva*, *H. sulcata*, *H. punctata*, *I. gibbosus* and *R. sanguineus s.l.* [8]. In another study in tick infested sheep, collected ticks belonged to the following species: *I. gibbosus*, *R. sanguineus*, *D. marginatus*, *H. parva* and *H. sulcata* [9]. However, *Borrelia burgdorferi s.l.* (*B.afzelii* and *B. garinii*) has been reported in *Ixodes* spp. ticks collected only from birds in Greece [10], to the authors knowledge.

Several mammalian species can be infected by *B. burgdorferi*, whilst some of them, such as rodents, insectivores, hares and deer, act as an important reservoir. Additionally, migratory birds are major participants of the transmission cycle, spreading the infected ticks into new territories [11]. Life and transmission cycle dynamics of the infected ticks can also be influenced by the climatic change, a critical issue facing humanity which contributes to the increased tick abundance, survival rate and host availability and significantly impacts the incidence of LB [12]. However, the impact of climatic variability on LB incidence is much more complex than its effect on distribution and population density of vectors as LB is only the end product of a complex chain of interactions between environment and human [13]. From this point of view gaining insight and identifying common patterns and drivers of zoonotic diseases is critical. Farm animals including sheep may be used as a sentinel of the interplay between *Borrelia* species, host and altered environment due to the climatic change. This reciprocal relationship between ecology and society has contributed to the emergence of new diseases as well the re-emerge of known ones to new geographical regions.

Vector-borne diseases represent the 17% of the total human infectious diseases, leading to more than 700,000 deaths globally every year [14]. LB is endemic, and foci are geographically distributed across north-western, central and eastern Europe, the United States and the Asian forestry regions [15]. LB is the most frequent vector-borne disease in humans in Europe, with a steadily increasing incidence of up to 360,000 cases over the last two decades [16]. In western Europe incidence of LB is equating to ~232,125 cases per year [17]. The higher prevalence was reported in Sweden, Germany, Austria, Belgium, Czech Republic, Slovenia and in other Baltic Rim countries.

In Greece, there are limited data on the prevalence of LB in humans [17]. Among the first cases of *B. burgdorferi* in Greece, one was reported in 1985 concerning only 1 seropositive individual and in two years later, two seropositives in the island of Crete [18]. In the Greek general population, the seroprevalence of *B. burgdorferi* antibodies was 1.1% in 1997 [19], 1.11% in a gipsy population of Attica in 1992 [20] and 3.3% in young males in 2000 [21]. 

Moreover, several recent studies reported the seroprevalence of *B. burgdorferi* antibodies in dogs of different regions of Greece. In a population of 2620 dogs in Greece, the seroprevalence of antibodies against *B. burgdorferi* was 2.23% in 2019, while double or triple tick-borne pathogen seropositivity was detected [22]. The same year in another study, *B. burgdorferi* antibodies were detected in only 1 out of 1000 dogs, with triple tick-borne pathogen seropositivity [23]. Finally, *B. burgdorferi* was not detected in a population of 1154 dogs living on Greek Ionian and Aegean islands including Crete in 2020 [24]. However, differences in the seroprevalence among these studies could be attributed to the different areas of dog residency, number of dogs sampled as well as the usage of different method for the detection of antibodies, and therefore different cut off values. The majority of the epidemiological studies in animals, refers to dogs and horses, with only few data available in sheep and cattle. The presence of *B. burgdorferi* DNA was detected, in a percentage of 6.2% of tick infested or not tick infested sheep in Tunisia [25]. The prevalence of *B. burgdorferi* antibodies among sheep was 23.8% in Egypt [26], 16.7% in Slovakia [27], 14.1% in Italy [28], 10% in Norway [29], while in Greece seroprevalence in sheep has not been reported to the authors best knowledge. 

LB is a multi-systemic disease affecting the nervous system, the heart and the joints [30]. The most common clinical manifestation in humans is erythema migrans, accompanied with flu-like symptoms in early stages [5,31]. Animals usually remain asymptomatic; thus, they may serve as a reservoir of the disease. However, LB has been described in cattle and horses presenting lameness, laminitis, swollen joints, chronic weight loss, fever and abortions [32,33,34]. A few cases of LB reported in sheep, with clinical manifestations including lameness, anorexia and poor body condition [35].

Hematological alterations associated with Lyme disease in humans have been firstly described in 1994 in the United states [36] and in Europe in 1988 [37], with the presence of thrombocytopenia and thrombocytopenic purpura, respectively. Similarly, anemia and thrombocytopenia, associated with *Borrelia persica* species were first reported in cats and dogs in Israel [38]. Furthermore in dogs with antibodies against *B. burgdorferi*, mild to moderate anemia, thrombocytopenia, leukocytosis, leukopenia, lymphopenia, neutrophilia and presence of band neutrophils were detected [39]. The hematological implication of the presence of antibodies against *B. burgdorferi* in sheep remains unknown. 

The purpose of the study was to identify possible variation in hematological variables associated with the presence of antibodies against *B. burgdorferi* in sheep. 

## 2. Results

A total number of 318 animals was tested. Out of the 162 samples meeting the inclusion criteria, 87 were negative for both IgM and IgG (group A), 9 were positive for IgM (group B), 18 were positive for IgM and IgG (group C), while 48 were positive for IgG (group D). 

**Figure 1:**
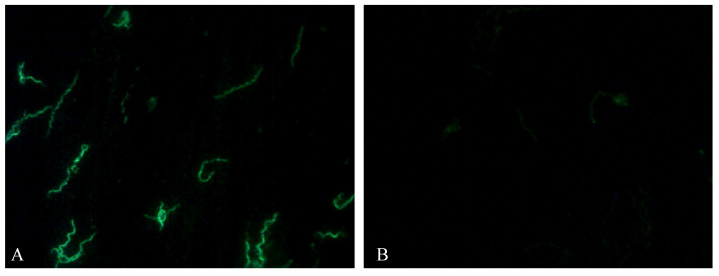
Picture of indirect immunofluorescence assay of serum sample from sheep. (**A**) Positive antibody reaction appears as bright sharp stained spirochetes. Sheep present IgG titer at 1:64 and (**B**) negative antibody reaction. Slides were observed using Nikon Eclipse E-400 fluorescence microscope, objective ×40.

A positive reaction appeared as bright sharp stained spirochetes was observed. The size, appearance and density were compared with the positive and negative controls reactions. Patterns of reactivity different than that seen in the positive control was considered non-specific, which means negative, as per manufacturer’s instructions. Positive and negative antibody reactions are shown in Figure 1.

**Figure 2:**
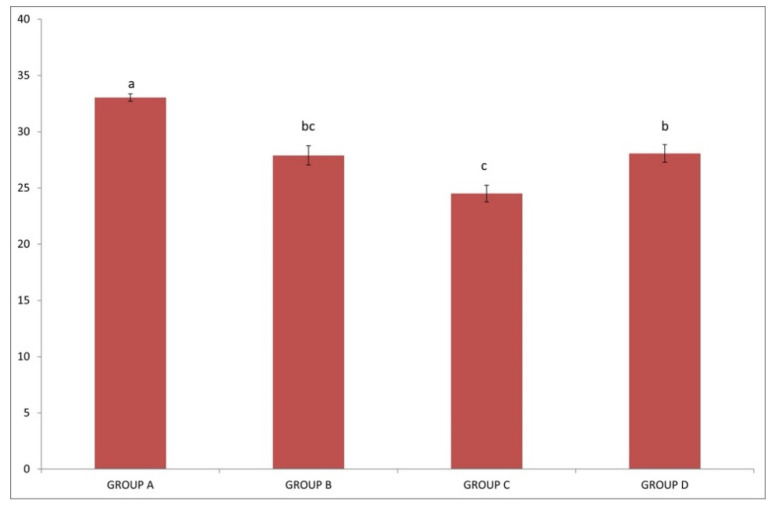
Each graph bar represents the average Packed Cell Volume (PCV) value of sheep in group A (IgM−/IgG−), group B (IgM+/IgG−), group C (IgM+/IgG+) and group D (IgM−/IgG+). Different lowercase letters a, b, c above each graph bar, are indicative of significant difference between groups.

Average Packed Cell Volume (PCV) (Figure 2) was significantly higher in group A (IgM−/IgG−) compared to all other groups (*p* < 0.05). The lowest value was recorded in group C (IgM+/IgG+) and was significantly different from those in groups A (IgM−/IgG−) and D (IgM−/IgG+) (*p* < 0.05). No significant difference (*p* > 0.05) was recorded between groups B (IgM+/IgG−) and C (IgM+/IgG+) and between groups B (IgM+/IgG−) (IgM+/IgG−), and D (IgM−/IgG+).

**Figure 3:**
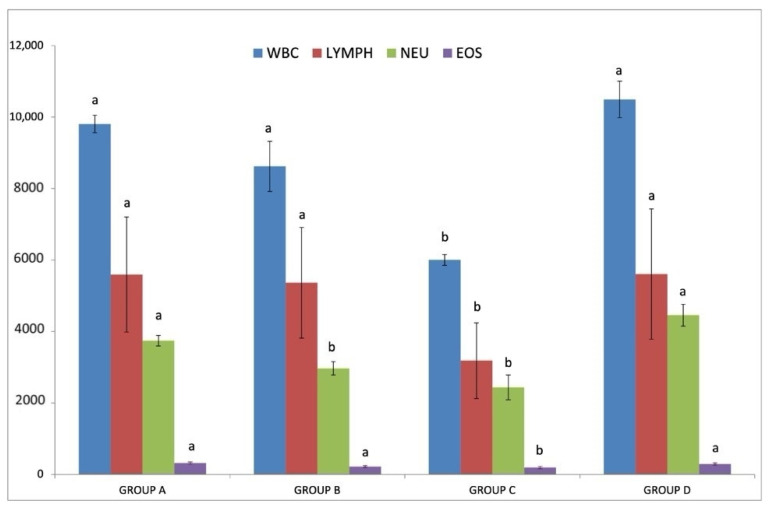
Each graph bar represents the average total white blood cells (WBC) (blue), neutrophils (NEU) (green), lymphocytes (LYMPH) (red) and eosinophils (EOS) (purple) counts in group A (IgM−/IgG−), group B (IgM+/IgG−), group C (IgM+/IgG+) and group D (IgM−/IgG+). Different lowercase letters a, b above each graph bar of the same color, are indicative of significant difference between groups.

As it is shown in Figure 3, average total white blood cells (WBC), neutrophils (NEU), lymphocytes (LYMPH) and eosinophils (EOS) counts were significantly lower in group C (IgM+/IgG+) in comparison with those in all other groups (*p* < 0.05); no significant difference was detected between the other three groups (*p* > 0.05). Band neutrophils were detected only in group C (IgM+/IgG+) and no basophil was detected in either sample. Monocyte counts were not significantly different among groups (means ± SE: 148.57 ± 14.95, 70.78 ± 41.55, 158.22 ± 39.92 and 138.35 ± 21.98, for groups A (IgM−/IgG−), B (IgM+/IgG−), C (IgM+/IgG+) and D (IgM−/IgG+), respectively; *p* < 0.05).

**Figure 4:**
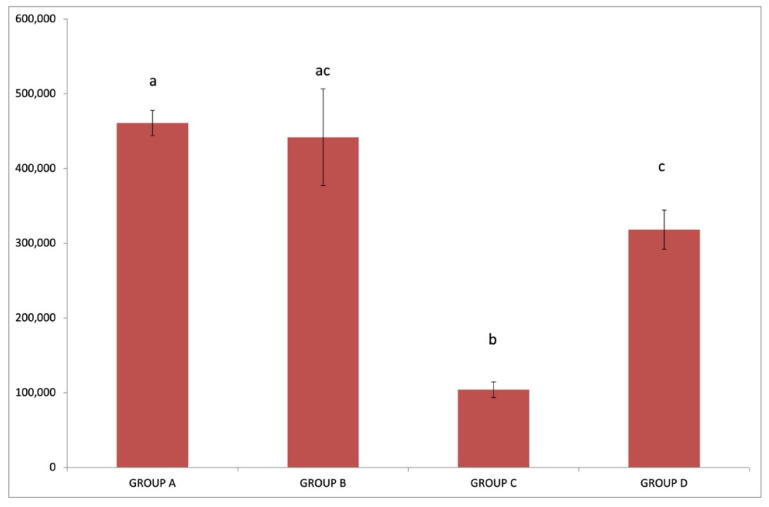
Each graph bar represents the average platelet (PLT) count value of sheep in group A (IgM−/IgG−), group B (IgM+/IgG−), group C (IgM+/IgG+) and group D (IgM−/IgG+). Different lowercase letters a, b, c, d above each graph bar, are indicative of significant difference between groups.

Average platelet (PLT) count (Figure 4) was significantly lower in group C (IgM+/IgG+) than those in all other groups (*p* < 0.05). The mean platelet values recorded in group D (IgM−/IgG+) were significantly lower compared to those in group A (IgM−/IgG−) (*p* < 0.05) but not in group B (IgM+/IgG−) (*p* > 0.05). The highest average platelet value was detected in group A (IgM−/IgG−) and was significantly different than those in groups C (IgM+/IgG+) and D (IgM−/IgG+) (*p* < 0.05).

Hematological findings of seropositive sheep included anemia, thrombocytopenia, leukopenia, neutropenia and lymphopenia, as well as leukocytosis, neutrophilia, lymphocytosis and eosinophilia. Blood smear microscopical examination revealed presence of anisocytosis, polychromatophilia and nucleated erythrocytes in anemic sheep indicative of regenerative anemia (blood loss and/or hemolysis) [40,41,42].

**Figure 5:**
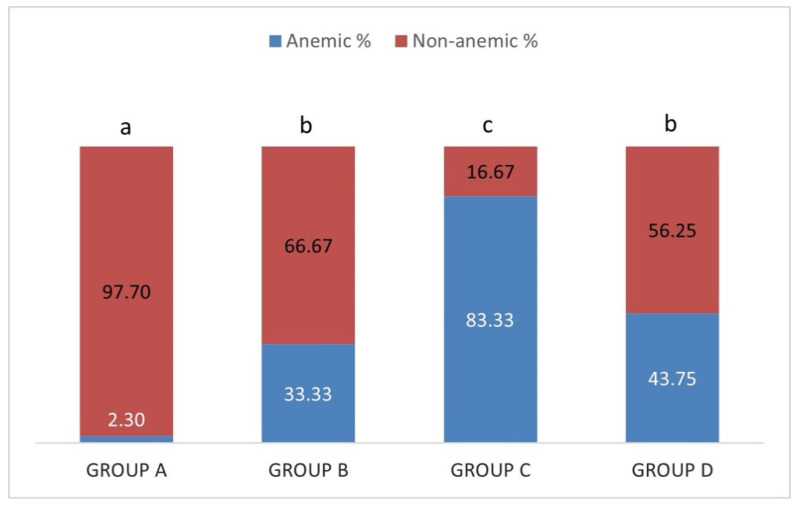
Each percentage stacked column represents the percentages of anemic (blue) and non-anemic (red) sheep in group A (IgM−/IgG−), group B (IgM+/IgG−), group C (IgM+/IgG+) and group D (IgM−/IgG+). Different lowercase letters a, b, c above each column, are indicative of significant difference between groups.

Regarding PCV value (Figure 5), 2.3% of the sheep in group A (IgM−/IgG−) were anemic, 33.33% in group B (IgM+/IgG−), 88.33% in group C (IgM+/IgG+) and 43.75% in group D (IgM−/IgG+). The percentage of anemic animals in group C (IgM+/IgG+) was significantly higher than all the other groups (*p* > 0.05). The percentage recorded in group A (IgM−/IgG−) was significantly lower (*p* < 0.05) compared to the other groups whereas no significant difference was detected between groups B (IgM+/IgG−) and D (IgM−/IgG+) (*p* > 0.05).

**Figure 6:**
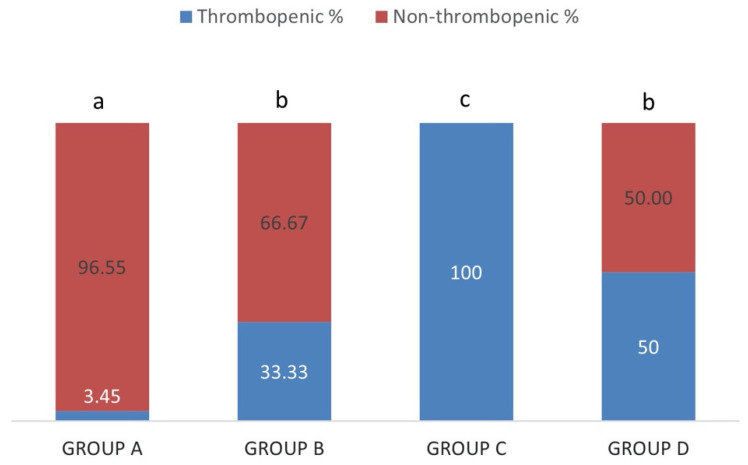
Each percentage stacked column represents the percentages of thrombocytopenic (blue) and non-thrombocytopenic (red) sheep in group A (IgM−/IgG−), group B (IgM+/IgG−), group C (IgM+/IgG+) and group D (IgM−/IgG+). Different lowercase letters a, b, c above each column, are indicative of significant difference between groups.

Thrombocytopenia was detected in 3.45% of the sheep in group A (IgM−/IgG−), 33.33% in group B (IgM+/IgG−), 100% in group C (IgM+/IgG+) and 50% in group D (IgM−/IgG+), as it is shown in Figure 6. The percentage of thrombocytopenic sheep was significantly higher in group C (IgM+/IgG+) and significantly lower in group A (IgM−/IgG−) in comparison with all the other groups (*p* < 0.05). The percentages between groups B (IgM+/IgG−) and D (IgM−/IgG+) were not significantly different (*p* > 0.05).

**Figure 7:**
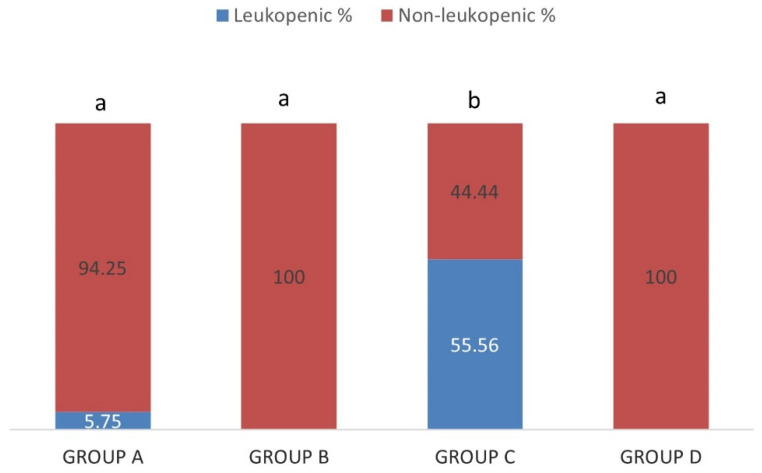
Each percentage stacked column represents the percentages of leukopenic (blue) and non-leukopenic (red) sheep in group A (IgM−/IgG−), group B (IgM+/IgG−), group C (IgM+/IgG+) and group D (IgM−/IgG+). Different lowercase letters a, b above each column, are indicative of significant difference between groups.

Out of the sheep of group A (IgM−/IgG−) and group C (IgM+/IgG+), 5.75% and 55.56% were leukopenic, respectively (Figure 7). No leukopenic animal was detected in groups B (IgM+/IgG−) and D (IgM−/IgG+). The percentage of leukopenic sheep in group C (IgM+/IgG+) was significantly higher than all the other groups (*p* < 0.05) whereas the difference of the percentages of leukopenic sheep among groups A (IgM−/IgG−), B (IgM+/IgG−) and D (IgM−/IgG+) was not significant (*p* > 0.05).

**Figure 8:**
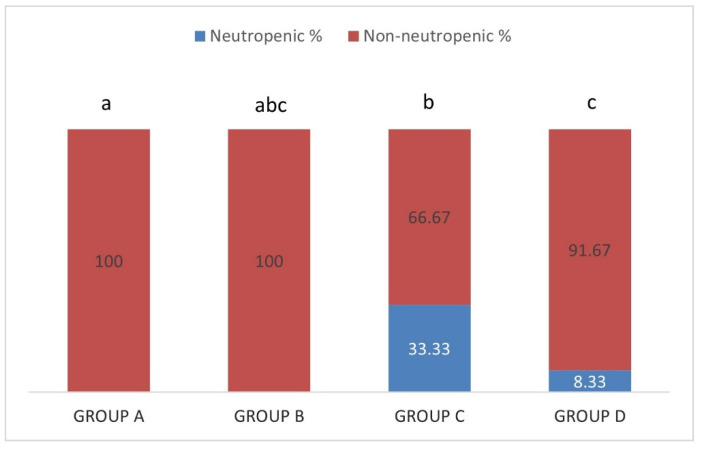
Each percentage stacked column represents the percentages of neutropenic (blue) and non-neutropenic (red) in group A (IgM−/IgG−), group B (IgM+/IgG−), group C (IgM+/IgG+) and group D (IgM−/IgG+). Different lowercase letters a, b, c above each column, are indicative of significant difference between groups.

Neutropenia was detected in 33.33% of the sheep in group C (IgM+/IgG+) and 8.33% in group D (IgM−/IgG+) (Figure 8). The percentages observed between groups A (IgM−/IgG−), C (IgM+/IgG+) and D (IgM−/IgG+) were significantly different (*p* < 0.05) whereas those in group B (IgM+/IgG−) were not significantly different than those in the other groups (*p* > 0.05).

**Figure 9:**
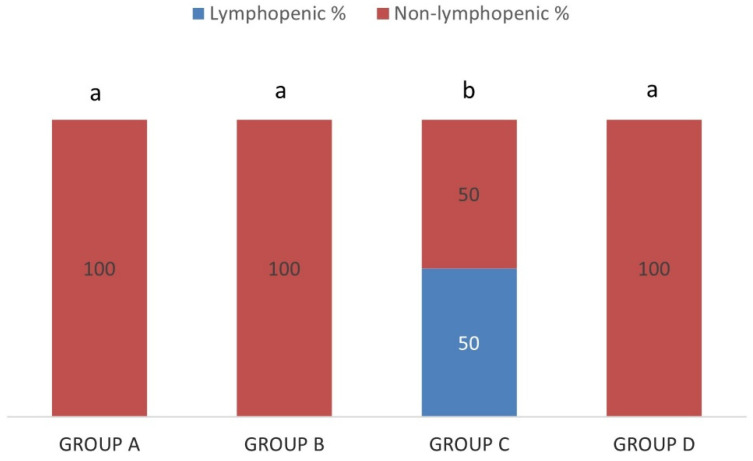
Each percentage stacked column represents the percentages of lymphopenic (blue) and non-lymphopenic (red) sheep in group A (IgM−/IgG−), group B (IgM+/IgG−), group C (IgM+/IgG+) and group D (IgM−/IgG+). Different lowercase letters a, b above each column, are indicative of significant difference between groups.

A percentage of 50% of sheep in the group C (IgM+/IgG+) presented lymphopenia but no lymphopenic animal was detected in other groups (Figure 9). The difference was significant between group C (IgM+/IgG+) and all other groups (*p* < 0.01).

Thrombocytosis was detected only in group A (IgM−/IgG−) in 1.15% of the sheep and the differences among groups were not significant (*p* > 0.05). 

A percentage of 22.92% of sheep in group D (IgM−/IgG+) presented leukocytosis whereas no leukocytosis was detected in the other groups. The difference was significant (*p* < 0.05) between group D (IgM−/IgG+) and groups A (IgM−/IgG−) and C (IgM+/IgG+), while no significant difference was detected among groups D (IgM−/IgG+) and B (IgM+/IgG−) (*p* > 0.05).

Neutrophilia was detected in 8.33% of the sheep in group D (IgM−/IgG+); this percentage was significantly higher than group A (IgM−/IgG−) (*p* < 0.05) but not with groups B (IgM+/IgG−) and C (IgM+/IgG+) (*p* > 0.05).

Lymphocytosis and eosinophilia were detected in 2.08% of sheep only in group D (IgM−/IgG+) and were not significantly different from all other groups (*p* > 0.05). 

## 3. Discussion

In the present study, the percentage of sheep found seropositive to *B. burgdorferi* was 23.58%. This result is in accordance with a previous study conducted in Egypt where seropositivity was found to be 23.8% in sheep with the highest infection rate detected in camels 47.8%, followed by 18.0% in goats, 16.0% in cattle and 10.9% in buffalos [26], while in China seroprevalence was up to 63.5% in sheep and goats [43]. The percentage detected here was unexpectantly high compared to the percentages of seropositivity recorded in dogs in Greece [22,23,24], since this is the first report in sheep. Sheep breeding in semi-extensive conditions and inadequate deworming program may be responsible for this important difference. Moreover, and most important, sampling was randomly performed from healthy dogs [22] while only tick infested sheep were included in the present study. 

The presence of both IgM and IgG antibodies was assessed, in order to identify recent or previous exposure to *Borrelia* species. Although antibody kinetics is unknown in sheep as in other animals, studies in humans revealed the presence of IgM antibodies at about 3 days after infection [44]. A weak antibody reaction was evidenced in a patient from the first day of infection in another study, which became stronger within a week [45]. Regarding IgG antibodies, they became apparent in low concentrations 1 week after infection which were increased within 4 weeks post infection [45]. Both IgM and IgG antibodies, remain detectable for months even years after infection [46,47]. The presence of IgM antibodies in patient effectively treated for the disease could be perceived as reinfection; however, there is a lot of skepticism on the real potential of serology to differentiate reinfection from persistent infection [48].

Based on the above data, it seems that sheep belonging to group B (IgM+/IgG−) are more likely to be in the early stage of infection, since IgM antibodies are usually present within short time after initial infection [49]. Therefore, a clear distinction on the stage of the infection of sheep in group C (IgM+/IgG+) and group D (IgM−/IgG+) is not feasible. However, a recent infection or reinfection is rather unlikely to occur in group D (IgM−/IgG+). 

Depending on the limit of detection of each method, false-negative results for both antibodies can occur during the first weeks of infection. In the present study, IFA assay was performed as an accurate and highly sensitive method for detecting antibodies against *B. burgdorferi* [50]. Although Western blot is considered the gold standard technique for LB confirmation due to its greater specificity, it is less sensitive than IFA especially in low IgG and IgM concentrations [50].

The production of specific antibodies against *Borrelia* is the main mechanism of host immune response. Despite the strong humoral response, the bacteria can survive for a long time in human [51]. Unraveling the mechanisms of *Borrelia* persistence is challenging. This long term presence of *Borrelia* in tissues implies that immune system interacts with bacteria antigens, recursively [52] and this is reflected in antibody kinetics.

False-positive antibodies against a certain *Borrelia* species may appear likely due to cross-reaction within the same bacterial genus (*Borrelia* complex known to cause Lyme disease and relapsing fever). As members of the *Borrelia* genus share common antigens with one another and with other spirochetes, cross-reactivity is expected [53]. False-positive results can occur due to cross-reaction with *Ehrlichia* and *Babesia* species as well as *Helicobacter pylori* [46]. In the present study, cross reactions cannot be ruled out since the incriminated pathogens in humans, i.e., *Rickettsia*, *Babesia*, *Leptospira* also infect sheep triggering immune response. The first two hemoparasites were neither cytologically or serologically detected in sheep included in this study, while *Leptospira* is rather infrequent in Greece and mainly associated with abortions [54], although it cannot be excluded. Moreover, farmers did not report severe rodent infestation. 

False positive test results may be due to cross-reacting antibodies to *B. burgdorferi* in patients with autoimmune disease such as systemic sclerosis and systemic lupus [46]. *Borrelia* employs several mechanisms to escape immune system, provoke chronic inflammation and trigger autoimmunity. Certain *Borrelia* species are found to escape complement system [55] while others alter the expression of surface proteins as a way to persist and establish chronic infection [56]. Dissemination of the spirochete among different tissues is achieved by its adhesion in host proteins, such are proteoglycans, integrins and several glycoproteins of cell or extracellular matrix [57]. Extracellular matrix is protective for *Borrelia* and may favor chronic inflammatory processes [58]. Finally, autoimmunity can be evoked by the mechanism of molecular mimicry, as sequence similarities between *Borrelia* and host tissue antigens may result to cross-activation of autoreactive lymphocytes by *Borrelia* antigens [59]. 

Generally, host antigenic stimulation by *Borrelia* induces gene expression of cytokines, chemokines, adhesion molecules and other immunomodulatory or immunosuppressive factors mediated by NF-kB and other transcriptional factors [60]. Especially, IL-10 suppresses the immune system, macrophages activation and production of proinflammatory mediators, allowing spirochete proliferation in vitro [61]. Except for cytokine induced suppression, *B. burgdorferi* was found to present cytopathic effects, killing B and T lymphocytes in cell cultures after active attachment, invasion through endocytic pits into vacuoles [62]. This ability of *B. burgdorferi* to invade and kill lymphocytes may be a possible explanation of lymphopenia in the 50% of sheep in group C (IgM+/IgG+). Similarly, immune suppression was reflected and in other white blood cells with the presence of neutropenia in sheep of group C (IgM+/IgG+) and group D (IgM−/IgG+). 

The lower mean count of WBC, as well as of neutrophils and lymphocytes, were decreased in sheep of group C (IgM+/IgG+), while the higher mean value of WBC and neutrophils, were detected in sheep of group D (IgM−/IgG+). The presence of normal or decreased WBC and mainly lymphopenia are in accordance with other studies in human [45,63,64] and dog [39] infected with *B. burgdorferi*. Likewise leukopenia was also detected in dogs and cats infected with *B. hispanica* [65,66]. On the contrary, monocyte count was not significantly different among groups. Relatedly, monocytes have found not to be susceptible as they effectively destroy *Borrelia* by phagocytosis [62]. Increased WBC count due to increased granulocyte and lymphocyte count was observed only in group D (IgM−/IgG+) and could be attributed to other pathologies probably not directly associated with the pathogenetic mechanisms of *Borrelia* in sheep with a relatively past exposure. However, in the total population of sheep with antibodies against *Borrelia* the most prominent features in WBCs are lymphopenia and absence of neutrophilia in the vast majority of sheep. The presence of normal total WBC count is a typical finding in human LB and supports differentiation of *Borrelia* infection from other bacterial infections where leukocytosis and increased polymorphonuclear cells are characteristically observed [63,64].

Anemia was observed in all groups with antibodies against *B. burgdorferi,* as well as in all tick infested sheep sampled in the present study due to a certain extent to tick infestation itself [67]. Erythrocytes attachment to glycosaminoglycans of *B. burgdorferi* was found to induce hemagglutination [68]. The destruction of erythrocytes may be a result of *Borrelia* hemolysin involvement. A hemolytic activity associated with *B. burgdorferi* has been detected, as certain *B. burgdorferi* strains produce oxygen-labile hemolysin enzymes with highest activity against horse erythrocytes, followed by bovine, sheep, and rabbit erythrocytes [69]. In the present study, anemia was more likely regenerative either due to blood loss or hemolysis that could be attributed to the above reported mechanisms [40,41,42]. Additionally anemia was evidenced in studies in human [70] and dogs with *B. burgdorferi* infection [39].

Finally, thrombocytopenia was detected in all sheep of group C (IgM+/IgG+) as well as in a considerable percentage of sheep belonging to other groups with antibodies against *B. burgdorferi*. This decrease of the number of PLTs may be attributed to increased platelet consumption or destruction rather than diminished production. In previous studies in human [36,71,72] and mice [73], normal number of megakaryocytes with absence of morphological abnormalities were observed, in bone marrow biopsies. More specific, spirochetes binding to integrins αIIbβ3, glycoproteins of the platelet membrane results in platelet activation and subsequent cell damage and removal from the circulation [36,74,75]. Autoimmune reactions have been proposed as a possible pathogenetic mechanism of thrombocytopenia, however this is a non- anonymously endorsed hypothesis [71,76].

Further studies are required to elucidate the underlying pathogenetic mechanisms of *Borrelia* including the impact on the immune and hemopoietic systems and the possible reflection of this impact in blood variables. *B. burgdorferi* is a common pathogen in both human and animals. From a “One Health” perspective, the presence of antibodies against *B. burgdorferi* in the present study is indicative of the sheep exposure to the pathogen. This raises concern about the dynamics of the circulation of *B. burgdorferi* in the area as sheep act as maintenance hosts for tick populations and potential spreaders of infected ticks and, consequently, tick-borne pathogens among humans and animals.

## 4. Materials and Methods

### 4.1. Sample Size

Prior to the onset of the study the minimum required total sample size was calculated using General Linear Multivariate Model with Wilks Likelihood Ratio procedure at the GLIMMPSE software (http://glimmpse.samplesizeshop.org/) [77]. The type I error rate was set at 0.05; the desired detectable difference in total white blood cell counts among groups was set at 10%, and the standard deviation of 10%. The means scale and variability factors were set at 1. The results of the analysis revealed that a minimum sample size of 24 animals (six per group, Power = 0.885) was required. 

### 4.2. Inclusion Criteria

A total number of 318 sheep of Chios breed from 6 farms in Greece was enrolled for the study. Animals included for the study based on (a) presence of ticks (b) deworming treatment performed the last 2 months. Animals were excluded based on (a) presence of other ectoparasites such as fleas and lice (b) serological or cytological evidence of concurrent tick-borne infections (*Anaplasma phagocytophilum*, *Babesia* sp., *Theileria* sp., *Rickettsia* sp.). If any animal was found positive to any of these tick-borne pathogens, it was excluded from the study.

### 4.3. Blood Sampling

Blood samples were collected by jugular venipuncture into plein and EDTA coated vacuum tubes (BD, Franklin Lakes, NJ, USA) for serum retrieval and whole blood, respectively. All samples were transferred on ice, avoiding direct contact with the tubes, to the Diagnostic Laboratory, Faculty of Veterinary Medicine, School of Health Sciences, University of Thessaly, Greece, within 24 h.

### 4.4. Complete Blood Count

Packed cell volume (PCV) value was assessed by the microhematocrit method [78]. The red cell column height formed after centrifugation of the tube is representative of the PCV value. 

Blood smear microscopy was performed in each sample. Blood smear preparations were fixed using methanol and stained with Giemsa. Quantitative assessment of white blood cells (WBCs) and platelets (PLTs) was performed as previously described [79]. Briefly, the number of WBCs and PLTs was microscopically assessed and a validated equation was used to convert the detected value to the corresponding value of the ADVIA 120 hematology analyzer [79]. Differential WBCs count was performed manually in 200 leukocytes. Interpretation of the values was based on previously published reference intervals [80].

### 4.5. Indirect Immunofluorescence Antibody (IFA) Assay 

Serum separation was performed after blood clotting by centrifugation of the tube at 300× *g* for 10 min. Serum supernatant was transferred into plastic vials (Eppendorf Tubes^®^, Eppendorf AG, Hamburg, Germany) and stored at −20 °C pending analysis. IFA assay was performed for the detection of IgG and IgM antibodies against *Borrelia burgdorferi* in serum.

Commercially available slides coated with *Borrelia burgdorferi sensu lato* antigens (MegaFLUO^®^ BORRELIA ph. Horbranz, Austria), a rabbit fluorescein isothiocyanate-conjugated anti-sheep IgG and a rabbit fluorescein isothiocyanate-conjugated anti-sheep IgM (Sigma-Aldrich, St Luis, MO, USA). Titers ≥1:64 were defined as positive [81]. Slides were observed using a Nikon Eclipse E-400 fluorescence microscope (objective ×40).

### 4.6. Groups

Based on the results of the antibody detection assays sampled animals were allocated in four groups; (a) group A: sheep found negative for both IgM and IgG presence, (b) group B: sheep with IgM against *B. burgdorferi*, (c) group C: sheep with IgM and IgG against *B. burgdorferi*, (d) group D: sheep with IgG against *B. burgdorferi*.

### 4.7. Data Analysis

Differences in the blood cell counts (WBC, neutrophils, band neutrophils, lymphocytes, monocytes, eosinophils, basophils, platelets) and the PCV among the four groups were analyzed using the statistical software IBM SPSS 25. The normality of the data was evaluated with the Kolmogorov-Smirnov test and the homogeneity of variances with Levene’s test. One-way ANOVA was run to determine the significance of the differences among groups for each parameter that was normally distributed. Post hoc comparisons were done using Bonferroni test when equal variances were assumed and Tamhane T2 test when the variances were unequal. The results are expressed as means ± SE. Chi-square test was used to assess the significance of the differences among groups in the percentages of sheep of each group presenting each of hematological findings (anemia, thrombocytopenia, etc.) d using in Medcalc^®^ statistical software. All comparisons were done at a significance level of *p* ≤ 0.05.

## 5. Conclusions

The results of the present study indicate that the infection with *B. burgdorferi* causes significant alterations on the hematological profile of sheep. However, the stage of the disease cannot be assessed in accuracy, when complete blood count and serological tests are combined. From a clinical point of view, in cases with thrombocytopenia along with anemia, normal or decreased WBCs and mainly lymphopenia, the infection with *B. burgdorferi* should be included in the differential diagnosis especially when the sheep are parasitized with ticks. The relatively high seroprevalence of *B. burgdorferi* recorded at this study provides also evidence for potential increased risk for Lyme disease in humans in rural areas that are in contact with sheep.

## Data Availability

The data presented in this study are available on request from the corresponding author. The data are not publicly available due to further processing for other studies.
